# The Book World of Medicine and Science

**Published:** 1906-06-30

**Authors:** 


### The Book World of Medicine and Science.

The Operative Treatment of Prolapse and Retroversion of the Uterus. By J. Inglis Parsons, M.D., M.R.C.P., M.R.C.S. (London : John Bale, Sons and Danielsson, Ltd. 1905. Price 3s. 6d. net.)

The theme around which the chapters of this book are written has already been exploited by the author in various papers to the medical journals, and at some meetings of the medical societies. As a theme it is interesting, instructive, and important. It was no accident of observation, such as befalls the fortunate, which originated the line of treatment to which we attach the author's name; it was the outcome of patient study and logical deduction. Previous workers in the field have no reason to complain of any want of generous recognition of their work, although the writer maintains his own opinion on all points open to debate. A good example will be found on p. 58, in the explanation of the causes which determine the occurrence of retroversion or retroflexion. Not so ready an assent will be given to his division of the stages of prolapse. No mention is made of the curved axis of the human pelvis, which many hold to be the factor in the association of retroversion with prolapse. The evolution of the erect posture gets its full value, in the production of prolapse; only that other fact of evolution, the curved axis, fails to obtain recognition. The author says (p. 15) : "At the beginning of the first stage, relaxation of the utero sacral ligaments allows the fundus to fall back." His opinion calls for notice because of the great work which he has done in relation to the repair of the broad ligaments, which forms a most interesting feature in the volume. "When the uterus is dislocated the process is usually so gradual that there is never . . . sufficient stimulus to cause the effusion of lymph necessary to produce repair" (p. 31). To produce such effusion the author selected a solution of quinine in acid, which he injected into the utero-pelvic band of the broad ligaments, and he records his experience that "natural repair takes place by the formation of new fibrous material" (p. 51). The relation of this new operation to the surgical procedures in common use is temperately discussed; but the fact remains that a new light has been thrown on the whole class of cases to which the affix "ptosis" applies. It matters little whether the author's quinine solution may give place to some more efficient agent, the results are satisfactory and capable of extension. Lymph forms the new suspensory bands in the operation of ventrosuspension; it ought to play a not less important part in strengthening enfeebled natural ligaments. As might be expected, Dr. Parsons leans rather towards reconstruction than new construction in his surgical work, and his criticisms are based upon physiology. The book and plates are well got up; some repetitions might be avoided in a re-issue; and on p. 71, "the use of the constant curette" (for current) hardly expresses the author's meaning. The volume will repay a careful study.

Diabetes Mellitus : its Pathological Chemistry and Treatment. By Professor Carl von Noorden. English translation by Florence Buchanan, D.Sc., and J. Walker Hall, M.D. (Bristol : John Wright and Company. Pp. 211. Price 5s. net.)

This is the seventh number of von Noorden's series of monographs dealing with the disorders of metabolism and nutrition. No one can fail to recognise in it the presence of a master's hand. This remark applies equally to the discussion of the several doctrines associated with the problem of diabetes and to the scheme of practical measures outlined as necessary for the treatment of the disease. In connection with the latter it is noteworthy that the author declines to associate himself with the prevailing tendency to diminish considerably the dietetic stringency formerly universally practised. On the contrary, he urges that "more stringent . . . diabetic treatment should be accorded to slight than to severe cases" on the ground that here there is both more to be gained and more to be lost. Altogether, on the subject with which it deals, no better guidance for the practitioner could be selected than that offered by von Noorden's essay.

Radiography and the "X" Rays : By S. R. Bottone. (Whittaker and Company. Illustrations 55; pp. x.+203. Second Edition.)

Lucidity and accuracy are of first importance in a practical handbook. On both counts this book can be commended to the student engaged in experimental work in radiography and the construction of *x*-ray apparatus. The author has assumed only a minimum of previous knowledge of electricity and photography, and has spared no pains to explain the details of construction by illustrations. In an experimental text-book reference to radio-therapeutics is necessarily brief; but a caution against the dangers of "*x*-ray burns" and an account of the measures likely to prevent their occurreuce have not been omitted. The construction of high-frequency apparatus has also received attention in the present edition.

